# Cardiac abnormalities of Sudanese patients with Down’s syndrome and their short-term outcome

**Published:** 2009-04

**Authors:** Sulafa KM Ali

**Affiliations:** Department of Paediatric Cardiology, Sudan Heart Centre, Khartoum Erkaweit, Sudan

## Abstract

**Background:**

Congential heart disease (CHD) is an important cause of morbidity and mortality in patients with Down’s syndrome (DS).

**Methods:**

All patients with DS seen at the Sudan Heart Centre from July 2004 to November 2007 were included in the study. All patients were examined clinically and echocardiographically, and cardiac catheterisation was carried out in selected patients. All patients were prospectively followed up.

**Results:**

In the study period, 1 566 patients were evaluated for heart disease. Of these, 80 patients with DS were identified (5%). Their ages ranged from 15 days to 18 years. Cardiac abnormalities included atrioventricular septal defect (AVSD) in 38 patients (48%), with the complete form in 25, a partial form in seven, AVSD with intact atrial septum in one, and complex AVSD in four patients. In one patient there was AVSD with right atrioventricular valve malformation with severe valve regurgitation and functional pulmonary atresia. The other main lesions were ventricular septal defect (VSD) in 19 patients (23%) and tetralogy of Fallot (TOF) in five (6%).

Cardiac catheterisation was done in four patients with AVSD to measure pulmonary pressures and resistance, and in one patient with patent ductus arteriosus for device closure. Ten percent of the patients had Eisenmenger’s syndrome at the time of presentation. Only 15% of patients who were in need of surgery were operated on; all had an uneventful postoperative course and a good outcome at a mean follow-up period of one year

**Conclusion:**

The pattern of CHD in Sudanese patients with DS was comparable with that in the literature, including the rare occurrence of AVSD with intact atrial septum. In addition, we described an unreported association with right atrioventricular valve malformation. Although there was a significant delay in diagnosis and surgery, surgical results and short-term follow up were good.

## Summary

Down’s syndrome (DS) is frequently associated with congenital heart disease (CHD), which usually leads to significant implications for the patients and their families. The most common defects are atrioventricular septal defect (AVSD) and ventricular septal defect (VSD).^1^-^3^ There have been, to our knowledge, no studies that determined the frequency and short-term outcome of Sudanese children with DS and CHD.

## Methods

All children diagnosed clinically to have DS, with or without confirmation of chromosomal studies, were included in the study. Patients were seen at the Sudan Heart Centre from July 2004 to November 2007. Clinical and complete echocardiographic examinations were done for each patient. Follow up was arranged according to the primary cardiac diagnosis. Cardiac catheterisation was done in selected patients for the following indications:

● to measure pulmonary artery pressure and vascular resistance
● to delineate anatomy not clearly identified on echocardiography
● for treatment in patients with lesions amenable to therapeutic catheterisation.

Parents were counselled regarding the nature of CHD and its treatment, as well as the consequences of DS on the patient, and its implications on the course of CHD and its treatment.

## Results

In the study period we identified 80 patients with DS out of 1 566 patients (5%). Their ages ranged between 15 days and 18 years.

## Echocardiographic findings

Seven patients had a normal echocardiogram (9%), 39 patients had (AVSD) (48%), 19 had VSD (23%) and five had TOF (6%). Others had a variety of lesions, as shown in Table 1.

**Table 1:** Echocardiographic Diagnoses And Outcome Of Patients With Down’s Syndrome

*Diagnosis*	*No*	*%*	*Outcome*
Normal	7	9	
**AVSD**	38	48	
Complete AVSD	25		Three patients had complete repair with good results, six had Eisenmenger’s syndrome on presentation. One with severe failure to thrive was not offered surgery, 15 patients were awaiting surgery
Partial AVSD	7		One had Ebstein-like tricuspid valve, for elective surgery
Unbalanced AVSD	4		Not offered surgery
AVSD with doubleoutlet right ventricle	1		Not offered surgery
AVSD with no primum ASD	1		Waiting for surgery
**VSD**	19	23	
Isolated VSD	15		One patient had complete repair with good results, two had pulmonary artery band, 13 were awaiting surgery
VSD with mitral valve cleft	4		Waiting for surgery
TOF	5	6	Two had complete repair with good results, three were awaiting surgery
PDA	6	7	One had PDA catheter closure, five were awaiting surgery
Isolated mitral valve cleft	2	2.5	Waiting for surgery
Secundum ASD	2	2.5	For elective surgery/catheter closure
VSD/PS with small right ventricle	1	1.5	Not offered surgery, died
Total	80	100	

Of those with AVSD, 25 had complete, balanced AVSD (primum ASD, a large inlet VSD, a common atrioventricular valve and normal size ventricles), seven had partial AVSD (primum ASD, small/absent inlet VSD, two atrioventricular valve orifices and normal size ventricles), and four had unbalanced AVSD (primum ASD, a large inlet VSD, a common atrioventricular valve and hypoplasia of one ventricle). One patient had AVSD and a double-outlet right ventricle with the aorta anterior to the pulmonary artery.

**Fig. 1.**
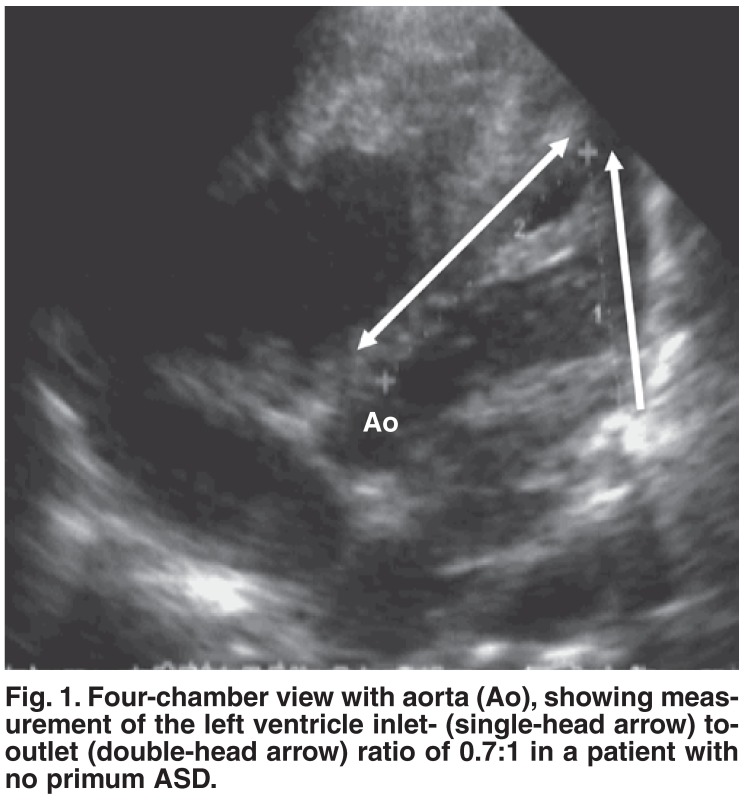
Four-chamber view with aorta (Ao), showing measurement of the left ventricle inlet- (single-head arrow) to-outlet (double-head arrow) ratio of 0.7:1 in a patient with no primum ASD.

**Fig. 2.**
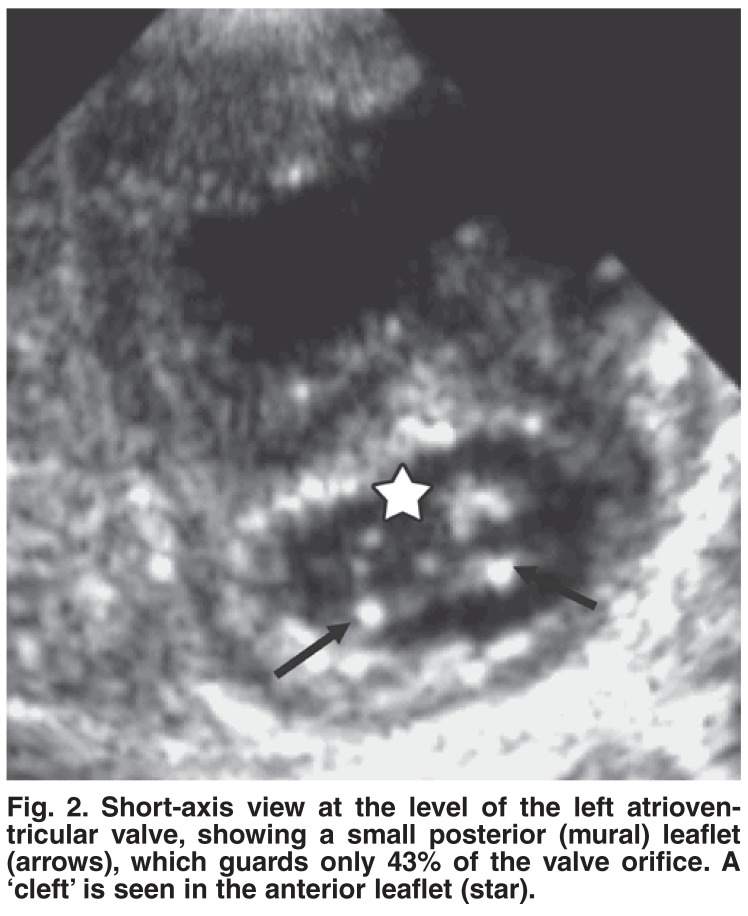
Short-axis view at the level of the left atrioventricular valve, showing a small posterior (mural) leaflet (arrows), which guards only 43% of the valve orifice. A ‘cleft’ is seen in the anterior leaflet (star).

One patient had AVSD with no primum ASD. In this patient the diagnosis of AVSD was based on the findings of:

● left ventricle inlet-to-outlet ratio of 0.7:1. The inlet distance was measured from the mitral valve posterior leaflet insertion to the left ventricle apex, and the outlet distance from the same point of the aorta to the left ventricle apex from the four-chamber with aorta view (Fig. 1).
● the ratio of the left atrioventricular valve guarded by the posterior leaflet of 43%. The posterior leaflet length was measured and compared to the circumference of the valve orifice from the short-axis view (Fig. 2).
● a common atrioventricular junction demonstrated from the subcostal view. In addition the patient had an inlet VSD and a ‘cleft’ of the left atrioventricular valve. The same criteria were used to differentiate AVSD from isolated mitral valve cleft.

**Fig. 3.**
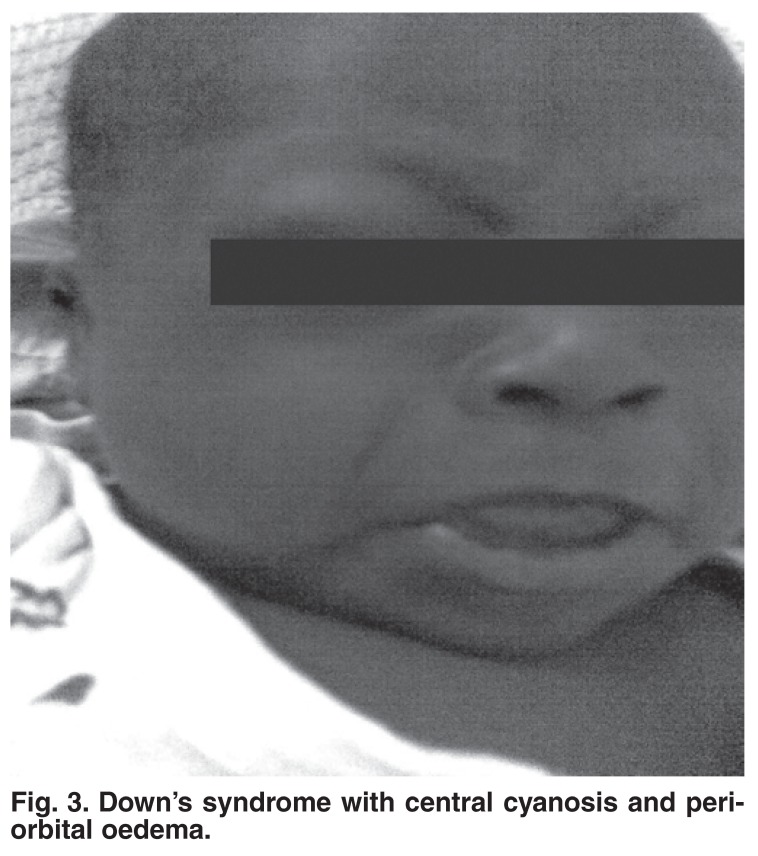
Down’s syndrome with central cyanosis and periorbital oedema.

**Fig. 4.**
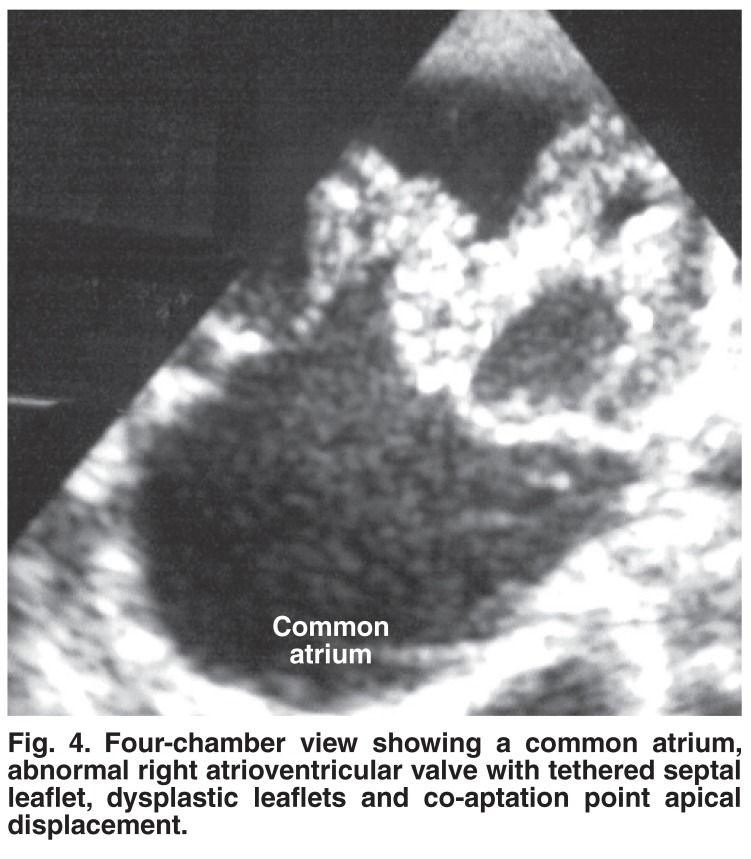
Four-chamber view showing a common atrium, abnormal right atrioventricular valve with tethered septal leaflet, dysplastic leaflets and co-aptation point apical displacement.

AVSD with abnormal right atrioventricular valve and functional pulmonary atresia: of those patients with partial AVSD, one neonate presented with severe cyanosis and right-sided heart failure (Fig. 3). Chest X-rays revealed a cardiothoracic ratio of 90%, a box-like configuration of the right atrium enlargement and oligaemic lungs. Echocardiography showed a common atrium with an abnormal right atrioventricular valve (Fig. 4). The septal leaflet of the valve was tethered to the interventricular septum and the co-aptation point was displaced apically with severe valve regurgitation that had a Doppler velocity of 2 m/s. Both leaflets looked thickened and dysplastic. No VSD was seen. There was a left atrioventricular valve cleft but no right-sided cleft. Decreased forward flow in the pulmonary artery without pulmonary stenosis was observed (functional pulmonary atresia).

Five patients underwent diagnostic cardiac catheterisation (Table 2). Three had complete AVSD and one had partial AVSD with low pulmonary vascular resistance. One patient had a successful PDA device occlusion using a 10/12 Amplatzer PDA occluder.

**Table 2:** Cardiac Catheterisation Findings In Down’s Syndrome

			Pulmonary artery pressure (mmHg)		Pulmonary vascular resistance (Woods units)		
*Age*	*Gender*	*Diagnosis*	*Room air*	*Oxygen*	*Room air*	*Oxygen*	*Procedure*
12 months	M	Complete AVSD	48	45	6	3	Diagnostic
16 months	M	Complete AVSD	50	55	4	3	Diagnostic
13 months	M	Complete AVSD	60	50	6	2	Diagnostic
7 years	M	Partial AVSD	30	7	0.5	0	Diagnostic
3 years	F	Large PDA	30	-	1	-	PDA device closure

## Outcome

Of the 80 patients with CHD, 52 needed surgery. Of these, eight were operated on (15%), six had complete repair and two had palliation. The cause of delay of surgery was low body weight (< 7 kg) in the majority of patients. Fourteen patients were not offered surgery: seven of these (10% of the total number of patients with CHD) had Eisenmenger’s syndrome on presentation, five had a functionally single ventricle and two had complex anatomy (Taussig Bing and Ebstein-like malformations). Ten patients were scheduled for elective surgery. All patients who had surgery had an uneventful immediate postoperative period and a good short-term outcome up to a mean follow-up period of one year.

## Discussion

This is the first report detailing echocardiographic abnormalities in Sudanese Down’s syndrome children and their short-term outcome. Generally, the spectrum of abnormalities seen in this cohort was comparable with that in the published literature.^2^,^3^ In addition, we diagnosed one patient as AVSD with no primum ASD, based on our previously reported observations on the echocardiographic features of AVSD.^4^ Measurements of the left ventricular inlet-to-outlet ratio of 0.7 and the left atrioventricular valve posterior leaflet guarding 43% of the valve orifice strongly supported the diagnosis of AVSD. In addition, these criteria helped us to differentiate patients with mitral valve clefts (in isolation or in association with VSD) from those with AVSD.

Many reports have described the occurrence of AVSD with intact atrial and ventricular septa, which used to be diagnosed on *post mortem* examination.^5^ Recently, there have been more reports on echocardiographic diagnosis of such cases by identifying a common atrioventricular junction, which can be helpful in diagnosing these cases.^6^

The association of Down’s syndrome with Ebstein malformation of the tricuspid valve is rare^7^ and to our knowledge there have been no reports on this association in the presence of AVSD. We suspect that our patient with partial AVSD and right atrioventricular valve deformity was in fact a case of Ebstein malformation because of the typical clinical and chest X-ray findings. Although the classical septal leaflet displacement and whip-like anterior leaflet were absent, the presence of a tethered septal leaflet and displaced co-aptation point supported the diagnosis. The presence of functional pulmonary atresia also favoured the diagnosis of Ebstein malformation, although this can be present in any case of severe tricuspid regurgitation. As we reported elsewhere, we observed a near five-fold increase in the frequency of Ebstein malformation in Sudanese patients, which reached 2.4% of all congenital heart disease, compared with the reported frequency of 0.5% in the western literature.^8^

Ten percent of patients with CHD presented with Eisenmenger’s syndrome, which indicated a delay in surgical referral of these patients. However, even in those who had early diagnoses, a large percentage were not operated on immediately, mainly due to their low body weight. With DS, the pulmonary vascular resistance can be irreversibly elevated as early as the first year, therefore delaying surgery beyond this age can be hazardous.^9^

Ethical issues regarding operating on patients with DS have been raised and discussed in many centres, and most people agree that DS patients should be offered surgery after careful counselling of their parents.^10^ In spite of their hypotonia and susceptibility to infection, our patients who had surgery had uneventful postoperative courses, as reported by others.^11^-^13^ In fact we noticed that the surgical outcome for our Down’s syndrome patients who had AVSD repair was much better than that of our non-Down’s syndrome patients. These observations are similar to those of Formigari *et al.*^14^

The patient who had a PDA catheter closure also had an uneventful course. Palliation of patients with AVSD with a pulmonary artery band is not widely accepted, especially when there is significant atrioventricular valve regurgitation. Therefore this procedure was done on only two patients.

## Conclusion

Our Down’s syndrome patients had, in general, a congenital heart pattern that was comparable with that of the literature, including the rare occurrence of AVSD with intact atrial septum. In addition, we described a new association with AVSD and right atrioventricular valve deformity. Although there was delay in diagnosis and surgery, the surgical results were good.
